# Stomatal Arrangement Pattern: A New Direction to Explore Plant Adaptation and Evolution

**DOI:** 10.3389/fpls.2021.655255

**Published:** 2021-04-30

**Authors:** Congcong Liu, Ying Li, Li Xu, Mingxu Li, Jianming Wang, Pu Yan, Nianpeng He

**Affiliations:** ^1^Key Laboratory of Ecosystem Network Observation and Modeling, Institute of Geographic Sciences and Natural Resources Research, Chinese Academy of Sciences, Beijing, China; ^2^College of Resources and Environment, University of Chinese Academy of Sciences, Beijing, China; ^3^Key Laboratory of Vegetation Ecology of Ministry of Education, Institute of Grassland Science, Northeast Normal University, Changchun, China

**Keywords:** stomatal traits, stomatal arrangement pattern, stomatal evenness, stomatal divergence, stomatal aggregation

## Abstract

The arrangement patterns of stomata on the leaf surface influence water loss and CO_2_ uptake via transportation and diffusion between stomata, the sites of photosynthesis, and vasculature. However, the quantification of such patterns remains unclear. Based on the distance between stomata, we developed three independent indices to quantify stomatal arrangement pattern (SAP). “Stomatal evenness” was used to quantify the regularity of the distribution of stomata based on a minimum spanning tree, “stomatal divergence” described the divergence in the distribution of stomata based on their distances from their center of gravity, and “stomatal aggregation” was used to quantitatively distinguish the SAP as clustered, random, or regularly distributed based on the nearest-neighbor distances. These three indices address the shortcoming of stomatal density that only describes “abundance” and may, collectively, have a better capacity to explore crop development, plant adaptation and evolution, and potentially ultimately enable a more accurate reconstruction of the palaeoclimate.

## Introduction

Stomata, formed by two guard cells, are valves that regulate the exchange of gasses between the leaf and the atmosphere. With this capacity, stomata have important effects on the global carbon and hydrologic cycles (Hetherington and Woodward, 2003). Currently, the measurement of stomatal morphology is a useful technique for taxonomists, physiologists, and other plant scientists, and there is rising interest among ecologists to integrate stomatal traits into process-based vegetation models (de Boer et al., 2016; Yan et al., 2017; Liu et al., 2020). Stomatal density, size, and their composite traits, including the stomatal pore area index and maximum stomatal conductance, have always drawn much research focus because of their roles in plant functioning and adaptation and as indicators of plant evolution (Franks et al., 2009; Sack and Buckley, 2016; Liu et al., 2018).

Stomatal arrangement patterns (SAPs) may play important roles in gas exchange (Harrison et al., 2020). Stomata need to occupy sufficient space to function properly, and a “one cell spacing rule” ensures neighboring stomata do not interfere with each other (Parlange and Waggoner, 1970; Franks and Casson, 2014). Therefore, stomata are separated by various shapes, sizes, and numbers of intervening cells (including epidermal cells and subsidiary cells) in a particular arrangement on the leaf surface (Carpenter, 2005). Stomata are developmentally coordinated with mesophyll (Baillie and Fleming, 2020) and xylem (Brodribb et al., 2017; Zhong et al., 2020), and various plants break this “rule” and produce stomata arranged in clusters, which reduce water loss from plant leaves due to neighboring stomata interfere with each other (Franks and Casson, 2014; Lehmann and Or, 2015). In comparisons of high and low clustering genotypes that have similar stomatal density and size, operational stomatal conductance was consistently lower in lines with highly clustered stomata, and stomatal clustering was shown to significantly reduce carbon assimilation (Dow et al., 2014; Supplementary Figure 1). Previous studies have clearly demonstrated that the arrangement patterns of stomata on the leaf surface require more research attention; however, ways to quantify SAPs have not yet been clearly defined.

Even under the same stomatal density, the patterns of stomatal arrangement could also be varied, thus stomatal density cannot characterize how stomata distributed on the leaf surface. Here, we developed new indices to characterize SAPs: stomatal evenness, stomatal divergence, and stomatal aggregation. The parameterization of SAPs may overcome the shortcoming of stomatal density (i.e., that it only describes “abundance”) and thus enable deep explorations of the evolution and adaptation of plants.

## Parameters to Characterize Stomatal Arrangement Patterns

**FIGURE 1:**
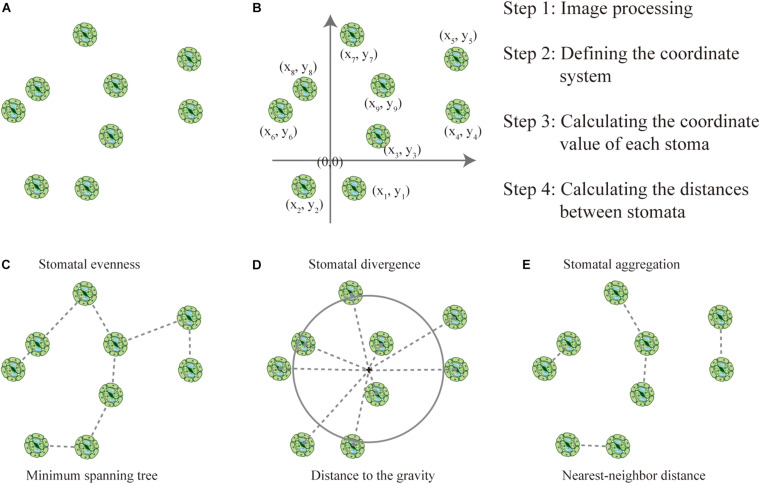
How to quantify stomatal arrangement pattern (SAP) based on the distances between stomata. **(A)** There is a total of nine stomata in the image, and stomatal density can be calculated as the ratio of the number of stomata to the area of the image. **(B)** The coordinates were established, and the locations of each stoma and the distances between stomata were quantified. **(C)** The minimum spanning tree (MST, dashed line) links the stomata. Stomatal evenness can represent the regularity of points along the MST. **(D)** Stomatal divergence can quantify how stomata diverge in their distances from their center of gravity. The center of gravity of the stomata is marked with a black cross, the gray dashed lines represent the distances of each stoma to the center of gravity, and the large gray circle represents the mean distance to the center of gravity. **(E)** Stomatal aggregation index was used to test the distribution type of stomata on the leaf surface (i.e., clustered, random, or regularly distributed) based on the nearest-neighbor distance.

The parameterization of stomatal arrangement patterns (SAPs) was based on the distance between stomata. The framework of this proposed method was based on the premise that stomatal size-related traits are conservative at the leaf level and would not influence the distance between stomata. Imaging is a critical step for the measurement of stomatal traits. Using the bottom-left corner or the center of the image as the origin, we could establish a coordinate system according to the scale of the image, whereby determining the coordinates of each stoma will enable the calculation of the distance between stomata (Figure 1). Please note that the position of the origin would not influence the distance between stomata. Distance between stomata is also an important parameter (Verboven et al., 2014), that can be measured using ImageJ^1^.

### Stomatal Evenness Index

Stomatal evenness was used to describe the regularity of the stomatal distribution on the leaf surface. In this process, the minimum spanning tree (MST) links all the stomata with the minimum sum of branch lengths (distance between stomata), and returns the *N* – 1 branches between the *N* stomata. As a first step, for each of these branches, its branch length is divided by the sum of all the branch lengths to obtain the partial distance (*PD*), defined as:

$$P\,D_{l}=\frac{D_{l}}{\sum_{l=1}^{N-1}D_{l}}$$

where *D* is the Euclidean distance between stomata, and the stomata involved is branch *l*. In the case of perfect regularity of stomatal distribution along the MST, all *PD*_*l*_ will be equal and all *PD*_*l*_ values will be 1/(*N* – 1). Conversely, when *PD*_*l*_ values differ among branches, the final index must decrease. Therefore, we compared *PD*_*l*_ values to 1/(*N* – 1). Finally, the stomatal evenness index (*SE*_ve_) was determined as follows:

$$S\,E_{\text{ve}}=\frac{\sum_{l=1}^{N-1}\min\left(P\,D_{l},\frac{1}{N-1}\right)-\frac{1}{N-1}}{1-\frac{1}{N-1}}$$

The term 1/(*N* – 1) is subtracted from the numerator and the denominator because there is at least one value of *PD*_*l*_ that is less than or equal to 1/(*N* – 1) regardless of the *N* value (Villéger et al., 2008). Therefore, *SE*_*ve*_ is unitless and is constrained between 0 and 1. A value of 1 is obtained when all *PD*_*l*_ values are equal to 1/(*N* – 1). Each image should contain more than three stomata to define an MST and then estimate *SE*_*ve*_.

### Stomatal Divergence Index

Stomatal divergence was used to describe the divergence in the distribution of stomata on the leaf surface. First, the coordinates of the center of gravity “G” of the *N* stomata contained in the image were calculated as follows:

$$\text{G}=\frac{1}{N}\,x_{i}$$

where *x*_*i*_ represents the coordinates of the *i*th stoma.

Second, for each of the *N* stomata, we calculated the Euclidean distance to this center of gravity:

$$d\,G_{i}=\sqrt{\sum {\left(x_{i}-G\right)}^{2}};$$

the mean distance of the *N* stomata to the center of gravity ($\bar{d\,G}$):

$$\bar{d\,G}=\frac{1}{N}\,\sum_{i=1}^{N}d\,G_{i},$$

the sum of deviances (△*d*) and absolute (△|*d*|) distances from the center of gravity, respectively, across the stomata:

$$△\,d=\sum_{i=1}^{N}\left(d\,G_{i}-\bar{d\,G}\right)$$

the sum of deviancand

$$△\,\left|d\right|=\sum_{i\,1}^{N}\left|d\,G_{i}-\bar{d\,G}\right|,$$

and finally, the stomatal divergence (*SD*_iv_):

$$S\,D_{\text{iv}}=\frac{△\,d+\bar{d\,G}}{△\,\left|d\right|+\bar{d\,G}}.$$

Values of *dG*_*i*_ are Euclidean distances and are thus positive or null values; hence, △*d* is bound between $\bar{d\,G}$and △|*d*|. Therefore, the addition of $\bar{d\,G}$to the numerator and the denominator ensures that the index ranges between 0 and 1. The index approaches 0 when many stomata are very close to the center of gravity and approaches unity when many stomata are very distant from the center of gravity.

### Stomatal Aggregation Index

The stomatal aggregation index was used to the measure of the degree to which the observed stomatal distribution departs from random expectation with respect to the distance to nearest neighbor. The nearest-neighbor distance of each stoma must follow the formula (Clark and Evans, 1954):

$$d=\frac{1}{2\,{\left(N/\mathrm{Area}\right)}^{0.5}}$$

where N is the number of stomata contained in the image, Area is the measured image area, thus the ratio of N to Area is stomatal density, and *d* is the theoretical nearest-neighbor distance.

The observed nearest-neighbor distance ($\bar{d}$) was calculated as follows:

$$\bar{d}=\frac{1}{N}\,\sum_{i=1}^{N}d_{i}$$

where *d_i* is the nearest-neighbor distance of the *i*th stoma.

The stomatal aggregation index (*SA*_*gg*_) represents the ratio of the observed nearest-neighbor distance to the theoretical nearest-neighbor distance.

$$S\,A_{\text{gg}}=\frac{\bar{d}}{d}$$

*SA*_*gg*_ values range from 0 to 2.15 (Clark and Evans, 1954), with values indicative of perfectly uniform (*SA*_*gg*_ value >1), random (*SA*_*gg*_ value = 1), and completely aggregated (*SA*_*gg*_ value < 1) patterns of distribution.

The significance of three stomatal patterning indices are summarized in Table 1, and the script (R statistical language) used to compute these three indices (*stomata_arrange* function) is available in the Supplementary Information.

**TABLE 1:** The range of stomatal patterning and their significance.

**Stomatal patterning**	**Range**	**Significance**
Stomatal evenness	0–1	Higher stomatal evenness means that neighboring stomata are less likely to interfere with each other, so that they function properly.
Stomatal divergence	0–1	Higher stomatal divergence means that stomata tend to be more globally not locally distributed. Higher stomatal divergence can shorten the diffusion distance of CO_2_, and cool leaf quickly.
Stomatal aggregation	0–2.51	The greater difference between stomatal aggregation and 1, the more stomatal distribution tends to be non-random.

## Relationships Between Stomatal Density and Stomatal Patterning

**FIGURE 2:**
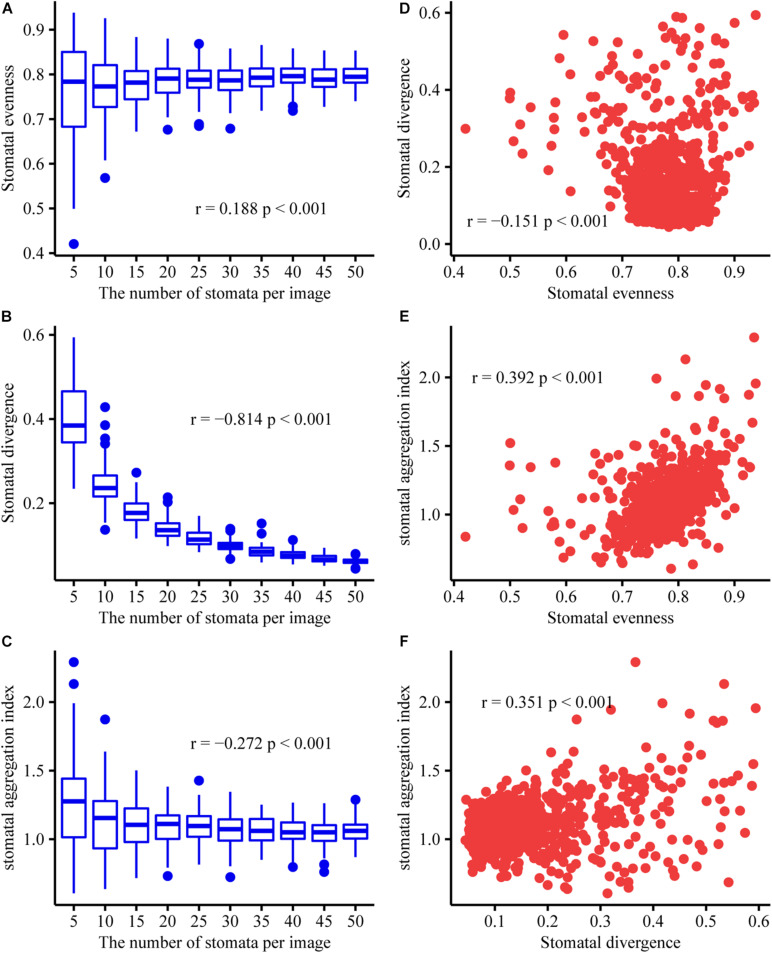
Relationships between stomatal density, stomatal evenness, stomatal divergence, and stomatal aggregation. Here, we used the number of stomata per image as a proxy for stomatal density. Pearson’s correlation coefficients (r) and the significance levels (p) are given in each subfigure. The data is randomly generated, and code is available in the Supplementary Information. **(A)** Correlation between stomatal density and stomatal evenness. **(B)** Correlation between stomatal density and stomatal divergence. **(C)** Correlation between stomatal density and stomatal aggregation. **(D)** Correlation between stomatal evenness and stomatal divergence. **(E)** Correlation between stomatal evenness and stomatal aggregation. **(F)** Correlation between stomatal divergence and stomatal aggregation. At the same stomatal density, the distributions of stomatal evenness, stomatal divergence, and stomatal aggregation were shown in Panel **(A–C)**, respectively.

To test whether the stomatal evenness, stomatal divergence, and stomatal aggregation indices are independent of stomatal density, we generated artificial stomatal arrangement patterns on the leaf surface. Given that a common maximum number of stomata per image (field of view) analyzed was usually below 50 (Fetter et al., 2019), we considered 10 stomatal-number levels (at intervals of 5). The coordinates of the stomata for each axis were also generated using a uniform distribution (i.e., all values had an equal chance of being selected) within a range of 100. For each stomatal-number level, the coordinates of each stoma were randomly generated, and then the stomatal evenness, stomatal divergence, and stomatal aggregation indices were calculated. This process was conducted 100 times. Our simulations using artificial datasets showed that the stomatal evenness and stomatal aggregation indices were weakly related to stomatal density; however, stomatal divergence was strongly correlated with stomatal density (Figure 2). Stomatal evenness, stomatal divergence, and stomatal aggregation were weakly correlated with one another, which indicates that they can represent the SAPs from different aspects. In addition, their range of variation decreased with stomatal density, which indicates that the adaptive significance of stomatal patterning is more important in species with a low stomatal density.

## Stomatal Patterning of Empirical Data

**FIGURE 3:**
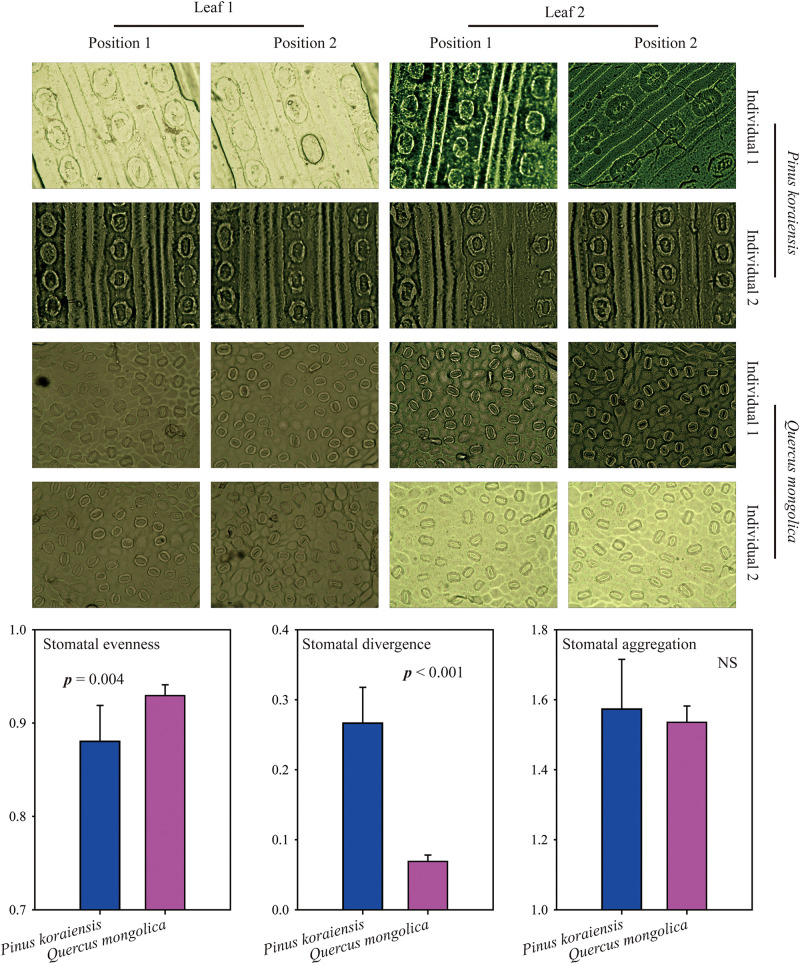
Images of stomata from *Pinus koraiensis* and *Quercus mongolica* and their comparisons on stomatal patterning. NS, Not Significant.

Here, *Pinus koraiensis* (coniferous tree) and *Quercus mongolica* (broad-leaved tree) were used to test the new indices to determine their repeatability. For each species, two individuals were selected; for each individual, two leaves were selected, and for each leaf, we randomly selected two locations to take the photomicrograph. All photomicrographs were measured from surface impressions of the abaxial leaf surface made with clear nail polish. We found that these new indices were relatively constant within species, and *SE*_*ve*_ of *Pinus koraiensis* was significantly lower than that of *Quercus mongolica*, while *SD*_*iv*_ of *Pinus koraiensis* was significantly higher than that of *Quercus mongolica*, indicating that stomata of *Quercus mongolica* were more evenly distributed, while stomata of *Pinus koraiensis* tended to be more globally distributed (Figure 3). Furthermore, we also found that *SD*_*iv*_, and *SA*_*gg*_ of *Angelica cartilaginomarginata* (forb) were significantly higher than that of *Phyllostachys heterocycla* (grass, Supplementary Figure 2), indicating that stomata of *Angelica cartilaginomarginata* were more globally and randomly distributed than that of *Phyllostachys heterocycla*. In addition, we selected herbarium specimens of nine species to quantify their stomatal arrangement patterns (Meeus et al., 2020), *SE*_*ve*_, *SD*_*iv*_, and *SA*_*gg*_ range from 0.90 to 0.93, 0.05 to 0.21, 1.34 to 1.72; respectively (Supplementary Figure 3). *SE*_*ve*_, *SD*_*iv*_, and *SA*_*gg*_ are independent from one another (Supplementary Table 1).

## Discussion and Future Directions of Stomatal Patterning

This work represents an important step toward a better understanding of stomatal arrangement patterns. Combining with stomatal density, size, and their SAPs (stomatal evenness, divergence, and aggregation), we might identify new candidate traits for crop development and exploring the adaptation and evolution of plants (Supplementary Figure 4). Therefore, it is important to investigate the following: (1) changes in these three new indices during ontogeny, (2) the coordination between these three new indices and other functional traits, (3) the response of these three new indices to global changes, and (4) the distribution of these three new indices at a large scale and their drivers. In addition, new indices could be derived from these three new indices, such as the ratio of abaxial and adaxial stomatal patterning for amphistomatous leaves, and how stomatal arrangements were involved in the evolutionary diversification of plants and development of amphistomaty should be the focus of future research (Muir, 2015; Haworth et al., 2018). Importantly, the quantification of the stomatal patterning of leaf fossils may assist in the reconstruction of paleoclimates (Beerling and Woodward, 1997). Furthermore, the indices developed in this study could also be further used to explore trichome patterning and other biological surface structures. Since we first developed indices to quantify the arrangement patterns of stomata, their effects on plant strategies have been overlooked, and the same is true for the remaining gaps in this research field. Thus, we call for additional efforts by researchers to explore the roles of stomatal patterning in plant functional ecology.

## Conclusion

We proposed a framework to quantify the arrangement patterns of stomata on the leaf surface, and originally developed three indices of stomatal evenness, stomatal divergence, and stomatal aggregation. These three indices might have important implications for our understanding of the adaptation and evolution of plants. Our framework has the potential to provide new insights that may enable scientists to better identify the roles of stomata in regulating CO_2_ and water exchange between the leaves and the atmosphere.

## Data Availability Statement

The original contributions presented in the study are included in the article/Supplementary Material, further inquiries can be directed to the corresponding author.

## Author Contributions

All authors planned and designed the research, wrote, edited, and revised the manuscript and approved the final version.

## Conflict of Interest

The authors declare that the research was conducted in the absence of any commercial or financial relationships that could be construed as a potential conflict of interest.

## Footnotes

1 https://imagej.net
