# Supplementary material for: Stomatal Arrangement Pattern: A New Direction to Explore Plant Adaptation and Evolution
Source: Front Plant Sci. 2021 Apr 30;12:655255. doi: 10.3389/fpls.2021.655255 (PMC8120035; doi:10.3389/fpls.2021.655255)
Supplement: Supplementary file 2 [file Data_Sheet_2.docx]

**Stomatal arrangement pattern: A new direction to explore plant adaptation and evolution**

Running title: How to quantify the stomatal arrangement patterns ?

Congcong Liu^1^, Ying Li^1^, Li Xu^1^, Mingxu Li^1^, Jianming Wang^1^, Pu Yan^1^ and Nianpeng He^1, 2, 3^*

1 Key Laboratory of Ecosystem Network Observation and Modeling, Institute of Geographic Sciences and Natural Resources Research, Chinese Academy of Sciences, Beijing 100101, China

2 College of Resources and Environment, University of Chinese Academy of Sciences, Beijing 100049, China

3 Institute of Grassland Science, Northeast Normal University, and Key Laboratory of Vegetation Ecology, Ministry of Education, Changchun 130024, China

*Correspondence author Nianpeng He (henp@igsnrr.ac.cn).

Tel.: +86-10-64889263

Fax: +86-10-64889399

**Table S1 Relationships between stomatal arrangement pattern (SAP) of nine species**

| **Variable 1** | **Variable 2** | **r** | **p value** |
| --- | --- | --- | --- |
| stomatal evenness | stomatal divergence | -0.31 | 0.42 |
| stomatal evenness | stomatal aggregation | 0.18 | 0.64 |
| stomatal divergence | stomatal aggregation | 0.16 | 0.68 |


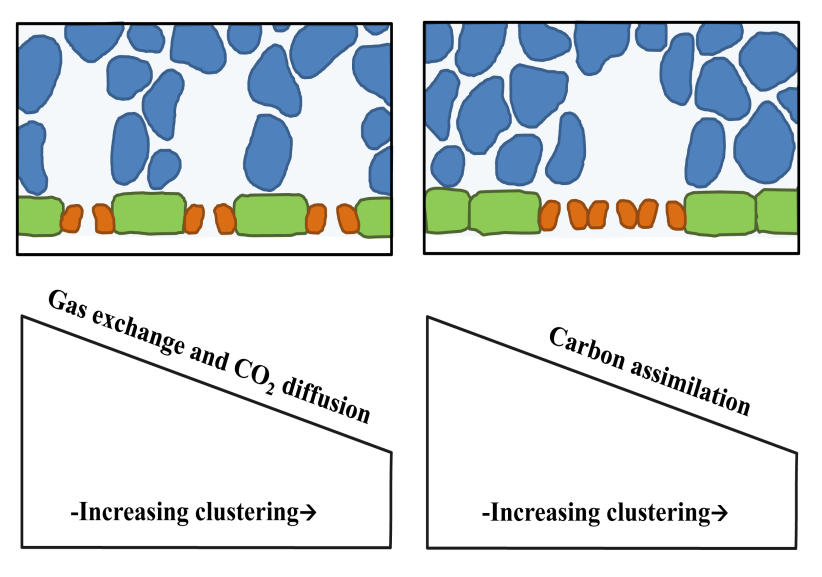


**Fig. S1 Stomatal arrangement pattern (SAP) can influence gas exchange and carbon assimilation between the leaves and the environment**

In the upper panels, blue represents spongy tissue, light green represents epidermal cells, and orange represents guard cells. The figure was adapted from the study by [Harrison *et al.* (2020](#_ENREF_10))

**
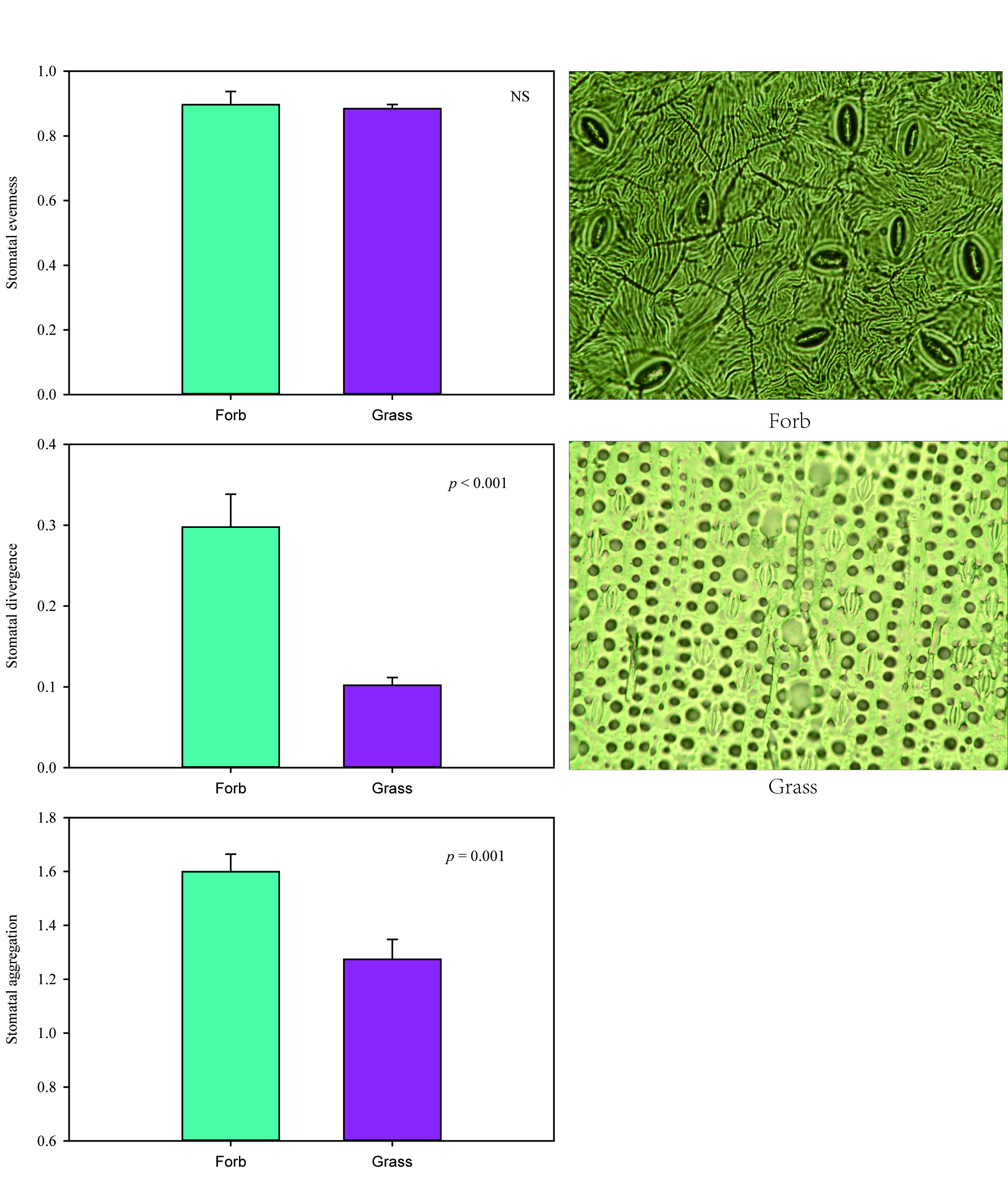
**

**Fig. S2 Stomatal arrangement patterns (SAPs) of Forb (*Angelica cartilaginomarginata*) and Grass (*****Phyllostachys heterocycla*).**

Nine replicates were conducted for each species.

NS, Not Significant.


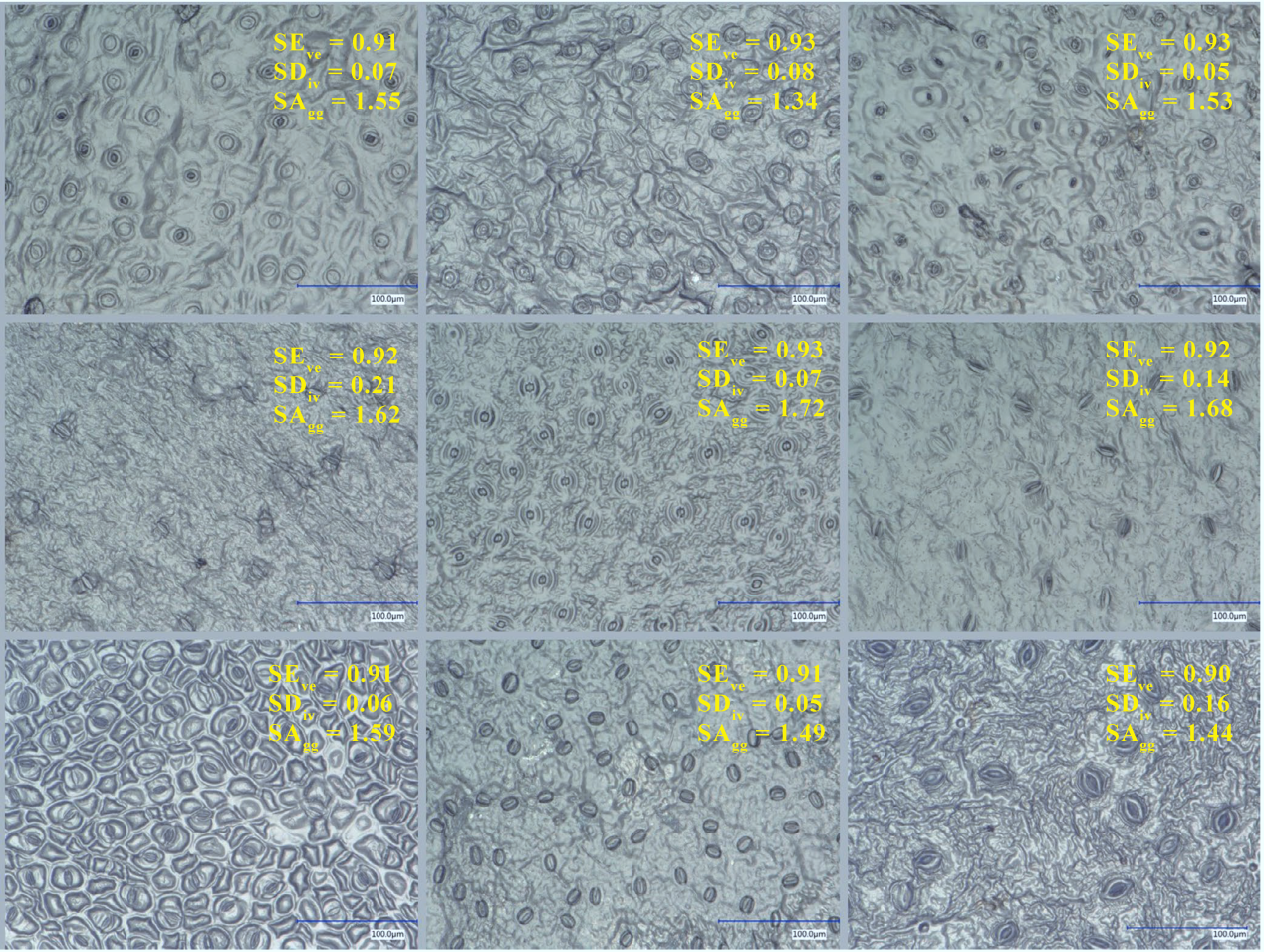


**Fig. S3 Stomatal arrangement patterns (SAPs) of nine species.**

Stomata microscope images are derived from [Meeus, et al. (2020](#_ENREF_16))

*SE*_ve_, *SD_iv_*, and *SA_gg_* are stomatal evenness, stomatal divergence, and stomatal aggregation indices, respectively

*Cola griseiflora*, *Carapa procera*, *Celtis mildbraedii*, *Garcinia punctata*, *Mammea africana*, *Petersianthus macrocarpus*, *Prioria balsamifera*, *Erythrophleum suaveolens* and *Trichilia gigliana* are sorted from top left to bottom right.

**Fig.** **S4 Stomatal arrangement pattern (SAP) should be a new dimension to explore plant adaptations**
