# Supplementary material for: Stomatal Arrangement Pattern: A New Direction to Explore Plant Adaptation and Evolution
Source: Front Plant Sci. 2021 Apr 30;12:655255. doi: 10.3389/fpls.2021.655255 (PMC8120035; doi:10.3389/fpls.2021.655255)
Supplement: Supplementary file 3 [file Data_Sheet_3.docx]

library(vegan)

#x=matrix(rnorm(10), nrow = 5)

?rnorm

xcor=runif(10,1,100)

ycor=runif(10,1,100)

x=cbind(xcor,ycor)

stomata_arrange<- function(x,image_area){

stomata_num=dim(x)[1]

PD=spantree(dist(x))$dist/sum(spantree(dist(x))$dist)

constant=1/( dim(x)[1]-1)

stomata_evenness=(sum(PD[PD<constant])+(dim(x)[1]-1-length(PD[PD<constant]))*constant-constant)/(1-constant)

######

distance_to_gravity=c()

for (i in 1:dim(x)[1]){

distance_to_gravity[i] =dist(rbind(x[i,],colMeans(x)))

}

mean_distance=mean(distance_to_gravity)

sum_deviance=sum(distance_to_gravity-mean_distance)

sum_abs_deviance=sum(abs(distance_to_gravity-mean_distance))

stomata_divergence=(sum_deviance+mean_distance)/(sum_abs_deviance+mean_distance)

stomatal_density=dim(x)[1]/image_area

theoretical_distance=1/(2*(stomatal_density^0.5))

nearest_neighbor_distance=c()

for ( ai in 1: dim(x)[1]){

nearest_neighbor_distance[ai]=sort(as.matrix(dist(x))[,ai])[2]

}

obseverd_distance=sum(nearest_neighbor_distance)/dim(x)[1]

stomata_aggregation=obseverd_distance/ theoretical_distance

return(cbind(stomata_num,stomatal_density,stomata_evenness,stomata_divergence,stomata_aggregation))

}

library(openxlsx)

#setwd(choose.dir())

#getwd()

setwd("C:/LCC/写作-lcc/气孔概念性论文/正文/投稿版本")

arrange=c()

for (i in 1:9){

data=read.xlsx("nine species .xlsx",i,rowNames=F)

stomata_arrange(data,342*256)

arrange=rbind(arrange,stomata_arrange(data,342*256))

}

cor.test(arrange[,5],arrange[,4])
